# Low Back Pain in Chinese Adults Aged 45 Years and Older: Trends, Drivers, and Projections, 1990–2040

**DOI:** 10.3390/healthcare14121692

**Published:** 2026-06-12

**Authors:** Samuhaer Azhati, Shuning Liu, Ruizhe Song, Mingchen Li, Yan Wei, Chang Liu, Huaichuan Zhang

**Affiliations:** 1 School of Education, Beijing Sport University, Beijing 100084, China; sam@bsu.edu.cn (S.A.); limingchen@bsu.edu.cn (M.L.); 2024011355@bsu.edu.cn (Y.W.); 2 School of Sport Science, Beijing Sport University, Beijing 100084, China; 2023013553@bsu.edu.cn (S.L.); c.liu@bsu.edu.cn (C.L.); 3 Sports Coaching College, Beijing Sport University, Beijing 100084, China; 2025010382@bsu.edu.cn

**Keywords:** low back pain, older adults, China, years lived with disability, risk factors, projection

## Abstract

**Background:** Low back pain (LBP) is a major cause of disability in later life. We aimed to assess the population-level burden, demographic and epidemiological drivers, GBD-defined risk attribution, and future trajectory of LBP among Chinese adults aged 45 years and older. **Methods:** Using population-level estimates from the Global Burden of Disease Study 2023 (GBD 2023), we analyzed incidence, prevalence, and years lived with disability (YLDs) among Chinese adults aged 45 years and older from 1990 to 2023. We assessed temporal trends, decomposed changes in burden, evaluated age–period–cohort patterns, quantified YLDs attributable to three GBD-defined risk factors—high body mass index, occupational ergonomic factors, and smoking—and projected burden to 2040 using Bayesian age–period–cohort models. **Results:** In 2023, population-level GBD estimates indicated that LBP accounted for 30.29 million incident cases, 71.54 million prevalent cases, and 7.90 million YLDs among Chinese adults aged 45 years and older. Compared with 1990, these numbers increased by 101.54%, 97.08%, and 96.11%, respectively, despite declining age-restricted age-standardized incidence, prevalence, and YLD rates. Expansion of the population aged 45 years and older was the main driver of the increasing absolute burden, whereas favorable epidemiological change offset part of this increase. High body-mass index showed the largest increase in attributable burden and was the only risk factor with rising age-standardized attributable YLD rates. Model-based projections suggested that age-restricted age-standardized burden would continue to decline through 2040. **Conclusions:** LBP remains a growing absolute burden among middle-aged and older adults in China despite declining age-restricted age-standardized rates. Future disability reduction will require integrated strategies combining risk-factor control, rehabilitation, functional support, and age-sensitive care.

## 1. Introduction

Low back pain (LBP) is the leading cause of years lived with disability (YLDs) worldwide, affecting more than 600 million individuals according to the Global Burden of Disease (GBD) 2021 study [1]. China possesses the world’s largest older population, with the number of individuals aged 60 years and older reaching 296.97 million in 2023 [2]. Furthermore, the country is undergoing rapid population ageing and faces substantial exposure to modifiable risk factors associated with LBP, including high body mass index (BMI), occupational ergonomic factors, and smoking [3,4,5]. The prevalence and disability burden of LBP increase markedly from midlife onward [6]. Adults aged 45 years and older are therefore a particularly policy-relevant population. We selected 45 years as the lower age threshold for three reasons. First, LBP burden begins to increase substantially from midlife onward, and age 45 captures the early stage of this increase rather than only later-life disability after retirement. Second, this threshold includes both late working life, when LBP contributes substantially to productivity loss, and the transition into older age, when multimorbidity and functional vulnerability amplify disability [7,8,9]. Third, 45 years corresponds to a standard 5-year GBD age-group boundary, enabling consistent extraction and comparison of age-specific estimates across the 1990–2023 period. Higher thresholds such as 50, 60, or 65 years would focus more narrowly on older age but would miss an important period for early prevention and functional preservation. Understanding the burden of LBP in this population is thus central to efforts to reduce disability and promote healthy ageing in China.

Despite this importance, several evidence gaps remain. Previous GBD-based studies have described the burden of LBP globally and in China, but most have focused on all-age populations, broad age groups, or single summary indicators rather than specifically examining Chinese adults aged 45 years and older over the full 1990–2023 period [1,6,10,11]. To our knowledge, no prior China-focused analysis has combined an age-restricted assessment of LBP burden in this population with decomposition of demographic and epidemiological drivers, age–period–cohort analysis, GBD-defined risk attribution, and future projection. This distinction is important because policy responses may differ depending on whether the increasing absolute burden is driven mainly by expansion of the population aged 45 years and older, ageing within this age range, or changes in age-specific epidemiology. In addition, limited evidence is available on the future trajectory of LBP and the changing contribution of modifiable risk factors among middle-aged and older adults in China.

Using GBD 2023 data, we examined the burden of LBP among Chinese adults aged 45 years and older from 1990 to 2023 and projected trends to 2040. Specifically, we assessed temporal trends and inflection points, quantified the relative contributions of demographic change and changes in age-specific epidemiology, examined descriptive age–period–cohort patterns, estimated the burden attributable to three GBD-defined risk factors—high BMI, occupational ergonomic factors, and smoking—and projected future burden using Bayesian age–period–cohort (BAPC) models. In the context of the Healthy China 2030 strategy [12], our findings are intended to support evidence-based priority setting for LBP prevention and care in China and may also offer insights for other countries undergoing rapid population ageing.

## 2. Methods

### 2.1. Data Source and Study Population

This study was a secondary analysis of publicly available aggregated estimates from the Global Burden of Disease Study 2023 (GBD 2023), extracted from the GBD Results Tool developed by the Institute for Health Metrics and Evaluation (IHME), University of Washington, Seattle, USA [13]. The tool is available through the Global Health Data Exchange at https://vizhub.healthdata.org/gbd-results/ (accessed on 10 April 2026) and is publicly accessible after free registration.

To improve reproducibility, data for the main burden analyses were extracted using the GBD Estimate option “Cause of death or injury”, rather than “Injuries by nature”, because low back pain is available as a cause under the former option. The following query parameters were used: location, China; cause, low back pain; years, 1990–2023; age groups, 45–49, 50–54, 55–59, 60–64, 65–69, 70–74, 75–79, 80–84, 85–89, 90–94, and 95+ years; sex, male, female, and both sexes; measures, incidence, prevalence, and years lived with disability (YLDs); and metrics, number and rate. For risk-attributable analyses, the GBD Estimate option “Risk factor” was used. The risk field was used to select high body mass index, occupational ergonomic factors, and smoking; the cause was set to low back pain; the measure was set to YLDs; and the metrics included number, rate, and percent, corresponding to attributable YLD numbers, attributable YLD rates, and population attributable fractions, respectively.

In the downloaded GBD files, standard fields include val, lower, and upper. The field val denotes the modeled point estimate for the selected stratum, while lower and upper denote the lower and upper bounds of the 95% uncertainty interval (UI), corresponding to the 2.5th and 97.5th percentiles of the ordered draw-level distribution, respectively. These values are not necessarily integers because they are modeled estimates rather than directly counted individuals. When the metric is “Number”, val represents the estimated number of cases or YLDs in the selected location-age-sex-year-measure stratum; when the metric is “Rate”, val represents the corresponding rate per 100,000 population; and when the metric is “Percent”, val represents the population attributable fraction. For example, a non-integer value such as 276,636.34 in the “Number” metric represents an estimated burden count for a specific GBD stratum, not a sampled number of individuals or a value per million population. Larger values, such as 80,479,694.83, similarly represent modeled burden counts for the corresponding selected stratum and may occur when broader or aggregate age categories are included in the downloaded output.

Although the GBD Results Tool may return additional aggregate age categories such as “55+ years”, “60+ years”, or “<70 years” depending on the selected age list or exported output, these aggregate categories were not used in the present analysis. After downloading, we restricted the analytic dataset to the eleven predefined 5-year age groups: 45–49, 50–54, 55–59, 60–64, 65–69, 70–74, 75–79, 80–84, 85–89, 90–94, and 95+ years. Any additional aggregate age categories in the downloaded file were treated as redundant outputs and excluded before calculation of truncated age-standardized rates, decomposition analysis, age–period–cohort analysis, and Bayesian age–period–cohort projection.

GBD 2023 synthesizes data from multiple sources, including population-based surveys, disease registries, health insurance claims, and published literature. For low back pain, national estimates are not simple counts from a single surveillance system but modeled estimates generated by integrating available epidemiological data, applying cross-walk adjustments where needed, and borrowing information across age, sex, location, and time when direct data are sparse. Therefore, the estimates may be influenced by the availability, representativeness, and quality of the underlying data sources, as well as by interpolation, extrapolation, and other modeling assumptions. These issues are reflected partly in the 95% UI provided by GBD. Non-fatal estimates are generated through Bayesian meta-regression using DisMod-MR, and risk-attributable burdens are derived from the comparative risk assessment (CRA) framework, which estimates burden based on exposure distributions, relative risks, and theoretical minimum risk exposure levels [14]. In GBD 2023, low back pain was defined as activity-limiting pain in the area on the posterior aspect of the body from the lower margin of the twelfth ribs to the lower gluteal folds, with or without pain referred into the lower limb, lasting for at least one day [15].

GBD estimates are not derived from a single individual-level dataset with one row per participant. Therefore, a conventional study sample size is not applicable to this analysis. The unit of analysis was the age-sex-year stratum, for which GBD provides modeled burden estimates and corresponding population denominators. The incident cases, prevalent cases, and YLDs reported in this study represent modeled national population-level estimates for China rather than the number of sampled individuals. Incident and prevalent cases refer to estimated burden counts, whereas YLDs represent years of healthy life lost due to disability.

We focused on Chinese adults aged 45 years and older because LBP burden increases substantially from midlife onward and because this age range captures both late working life and older adulthood, which are highly relevant to healthy ageing in China. All GBD estimates were reported with 95% UIs as provided in the GBD 2023 Results Tool. The UIs correspond to the 2.5th and 97.5th percentiles of the ordered draw-level distribution generated in the GBD estimation process. These draws represent posterior or model-simulation draws of burden estimates rather than randomly sampled individual patients.

### 2.2. Outcome Measures

We characterized LBP burden using three epidemiological indicators: incidence, prevalence, and YLDs. Incidence referred to the annual number and rate of new LBP episodes, capturing both first-ever and recurrent symptomatic episodes as defined in the GBD framework [14]. Prevalence referred to the number and rate of individuals living with LBP in a given year (point prevalence), reflecting the extent of non-fatal health burden. YLDs quantified health loss due to LBP and were estimated within the GBD framework by multiplying the prevalence of each severity-specific health state by its corresponding disability weight and summing across health states, with adjustment for comorbidity [14]. Disability weights range from 0 (no health loss, equivalent to full health) to 1 (complete health loss, equivalent to death) [16].

To enable comparisons over time while accounting for differences in age structure within the study population, we calculated age-restricted age-standardized rates, hereafter referred to as truncated age-standardized rates or truncated ASRs, per 100,000 population. Because this study focused on adults aged 45 years and older, age-specific rates were standardized using the GBD world standard population restricted to age groups 45–49 through 95+ years, with the corresponding standard population weights renormalized to sum to one within this restricted age range. Direct standardization was performed as follows [14]:

$$ASR=\frac{\sum_{i=1}^{N}a_{i}w_{i}}{\sum_{i=1}^{N}w_{i}}\times 100,000$$

where $a_{i}$ denotes the age-specific rate in age group *i*, and $w_{i}$ denotes the corresponding renormalized weight from the GBD world standard population restricted to the included age groups. Truncated age-standardized incidence rates, truncated age-standardized prevalence rates, and truncated age-standardized YLD rates were derived for each year from 1990 to 2023 and used as the primary indicators in subsequent trend analyses. These age-restricted rates are not directly comparable with standard all-age GBD age-standardized rates because the standard population weights were renormalized within the age range of 45 years and older.

### 2.3. Temporal Trend Analysis

To characterize temporal patterns from 1990 to 2023, we applied Joinpoint regression using the Joinpoint Regression Program (version 5.0.2; National Cancer Institute, USA) [17]. The model fits connected log-linear segments to the annual truncated ASRs and identifies time points at which the slope of the fitted trend changes [18]. Because the underlying estimates were modeled GBD point estimates, Joinpoint results were interpreted as exploratory descriptions of temporal patterns rather than definitive evidence of abrupt epidemiological shifts. The optimal number of joinpoints was determined by the Monte Carlo permutation test (4499 permutations; α = 0.05), with a maximum of five joinpoints permitted to balance model flexibility and overfitting given the 34 annual observations [19]. For each identified segment, we estimated the annual percentage change (APC), defined as the slope of the log-linear segment expressed as a percentage. The average annual percentage change (AAPC) over the full study period was calculated as a weighted geometric average of the segment-specific APCs, with weights proportional to the length of each segment [20]. Trends were classified as increasing or decreasing according to the sign of the APC or AAPC, with statistical significance inferred when the corresponding 95% confidence interval (CI) did not include zero.

The 95% CIs for APCs and AAPCs were generated by the Joinpoint models and are distinct from the 95% UIs accompanying GBD estimates. Because the Joinpoint and EAPC analyses were based on annual modeled point estimates, uncertainty in the underlying GBD estimates was not directly propagated into the trend detection or log-linear regression processes. Therefore, Joinpoint-derived APCs, AAPCs, and EAPCs should be interpreted as descriptive trend summaries of modeled GBD estimates rather than as analyses of raw surveillance data. Analyses were performed separately for truncated ASIRs, truncated ASPRs, and truncated age-standardized YLD rates, stratified by sex. Joinpoint-derived APCs and AAPCs were used as the primary trend indicators because they allow non-linear temporal patterns and changes in slope to be identified. EAPCs derived from simple log-linear regression were reported as supplementary overall trend summaries to facilitate comparison with previous GBD-based studies. Specifically, EAPCs were fitted to the annual truncated ASRs using the following model:

$$ln({\mathrm{rate}}_{t})=\alpha +\beta \times {\mathrm{year}}_{t}$$

where rate_t denotes the truncated ASR in year *t*, α is the intercept, and *β* is the slope coefficient. The EAPC and its 95% confidence interval (CI) were calculated as:

$$\mathrm{EAPC}=100\times \left[exp(\beta )-1\right]$$

$$95\%\;\mathrm{CI}=100\times \left[exp(\beta \pm 1.96\times SE_{\beta })-1\right]$$

EAPCs and AAPCs were interpreted as complementary rather than interchangeable summary measures because they are based on different modeling assumptions. AAPCs summarize Joinpoint-derived segmented trends using period-length weights, whereas EAPCs impose a single log-linear trend over the full study period. Therefore, EAPCs are not expected to be numerically identical to AAPCs or to endpoint-based average annual changes, particularly when temporal trends are non-linear.

### 2.4. Decomposition Analysis

Changes in the numbers of incident cases, prevalent cases, and YLDs between 1990 and 2023 were partitioned into three components—growth of the population aged 45 years and older, ageing within the ≥45 population, and epidemiological change—using the Das Gupta decomposition method [21]. Within this framework, the total count of each outcome is expressed as:

$$C=P\times \sum_{i=1}^{N}s_{i}\times r_{i}$$

where *C* is the total number of cases or YLDs among adults aged 45 years and older, *P* is the total population size within this age range, s_i_ is the proportion of the ≥45 population in age group *i*, and *r_i_* is the corresponding age-specific rate. The net change in *C* between the two endpoints is then additively decomposed as:

$$\Delta C=\Delta C_{population}+\Delta C_{ageing}+\Delta C_{epidemiology}$$

where each Δ*C* term isolates the effect of changes in one factor while holding the other two constant. To eliminate dependence on the order of factor substitution, we applied Das Gupta’s symmetrical replacement procedure, which averages contributions across all possible orderings and ensures that the three components sum exactly to the observed total change [21]. Population size and age structure were obtained from the GBD 2023 population estimates for China [22].

The decomposition was performed separately for each outcome (incidence, prevalence, and YLDs) and stratified by sex (male, female, and both sexes combined). For each component, we report absolute contributions (in numbers of cases or YLDs) and relative contributions (expressed as percentages of the total observed change). Because the three components may act in opposing directions, relative contributions can be negative or exceed 100% in magnitude: a positive value indicates that the component drove the outcome count upward, whereas a negative value indicates that it offset this trajectory.

### 2.5. Age–Period–Cohort Analysis

We applied the age–period–cohort framework to assess age, calendar period, and birth cohort-related patterns in LBP incidence, prevalence, and YLDs [23]. Annual case counts and population denominators from GBD 2023 were aggregated within each age-by-period cell, generating a table of 11 age groups (45–49 to 95+ years) and 7 calendar periods: six complete 5-year periods from 1990–1994 to 2015–2019 and one incomplete recent period covering 2020–2023. Because the most recent period contained only four years of available data, it was retained as an incomplete recent interval to preserve the complete study span and the most recent data window. Estimates for this final period were interpreted cautiously. Birth cohorts were derived from the midpoints of the age and period intervals, yielding 17 overlapping 5-year birth cohorts spanning approximately 1895 to 1975.

Estimation was implemented in R (version 4.3.0) using the analytical framework developed by Rosenberg and colleagues [24], which fits a log-linear Poisson model with population denominators as an offset and addresses the non-identifiability of the conventional age–period–cohort model by focusing on estimable second-order functions of the parameters. From this framework, we derived: (i) net drift, the overall annual percentage change in age-adjusted rates reflecting the combined linear effects of period and cohort; (ii) local drifts, the age-specific annual percentage changes; (iii) the longitudinal age curve, representing fitted age-specific rates in the reference cohort adjusted for non-linear period and cohort effects; (iv) period rate ratios (RRs) relative to the reference period centered at 2007.5 (i.e., 2005–2009); and (v) cohort RRs relative to the central reference cohort (1935). The central period and cohort were chosen as reference groups to provide stable and interpretable comparisons. Statistical significance of net drift, local drifts, and the relevant age–period–cohort deviations was assessed using Wald χ^2^ tests (two-sided *p* < 0.05). Analyses were performed separately for each outcome (incidence, prevalence, and YLDs) and stratified by sex (male, female, and both sexes combined). The APC analysis was intended to describe age-, period-, and cohort-related patterns rather than to identify independent causal effects. Because of the exact linear dependency among age, period, and cohort, the linear components of these dimensions cannot be uniquely separated without modeling constraints. Therefore, period and cohort RRs were interpreted as descriptive contrasts based on estimable functions, not as direct evidence of causal effects of calendar-time changes or birth-cohort characteristics.

### 2.6. Risk Factor Attribution

We examined the contribution of three GBD-quantified modifiable risk factors—high body mass index, occupational ergonomic factors, and smoking—to YLDs among Chinese adults aged 45 years and older. These risk factors were selected because GBD 2023 provides comparative risk assessment estimates for their attributable contribution to LBP YLDs. Thus, the present study did not test individual-level associations between these exposures and LBP; rather, it summarized population-level attributable burden estimated within the GBD CRA framework. Because LBP is evaluated in the GBD framework as a non-fatal condition, risk-attributable burden was assessed using YLDs only [14]. Age-, sex-, and year-specific attributable YLD numbers and population attributable fractions (PAFs) were extracted directly from the GBD 2023 CRA outputs [14].

In the CRA framework, attributable burden is estimated by combining the exposure distribution for each risk factor, the corresponding relative risk function for the LBP risk–outcome pair, and the theoretical minimum risk exposure level (TMREL) defined by GBD. Occupational ergonomic factors in GBD capture exposures such as heavy lifting, prolonged awkward postures, and whole-body vibration; high BMI reflects excess body mass relative to the GBD-defined minimum-risk exposure distribution; and smoking reflects exposure categories incorporated in the GBD smoking risk framework. Potential mediation and overlap among risks are handled within standard GBD CRA procedures to reduce double-counting when estimating attributable burden. These estimates should therefore be interpreted as population-level attributable YLDs under the GBD comparative risk assessment assumptions, not as direct individual-level causal effects estimated in the present study.

For adults aged 45 years and older, we additionally calculated age-restricted age-standardized attributable YLD rates, hereafter referred to as truncated attributable ASRs, per 100,000 population by direct standardization across the included age groups, using the corresponding weights from the GBD global age-standard population after renormalization within the restricted age range. We summarized attributable burden using annual attributable YLD numbers, truncated ASRs, relative contributions of each risk factor to the combined attributable YLD burden, and age-specific PAFs. Temporal trends in truncated ASRs were quantified using EAPCs derived from log-linear regression models of the form ln(rate) = α + β × year, with EAPC calculated as $100\times (e^{\beta }-1)$. A trend was considered statistically significant when the corresponding 95% confidence interval did not include zero [20].

### 2.7. Bayesian Age–Period–Cohort Projection

To project the future burden of LBP among Chinese adults aged 45 years and older, we fitted BAPC models to historical incidence, prevalence, and YLD data from 1990 to 2023 and generated annual model-based projections for 2024–2040. The projections assume that historical age–period–cohort structures and temporal patterns continue in broadly similar ways over the projection horizon. Formal back-validation or comparison with alternative projection models was not performed; therefore, the projections were interpreted as exploratory estimates for planning rather than definitive forecasts. The projection horizon was specified a priori to provide medium-to-long-term estimates extending beyond the Healthy China 2030 milestone while avoiding the greater uncertainty associated with longer extrapolations such as projections to 2050. The year 2040 represents a 17-year projection horizon after 2023, approximately half of the 34-year historical observation period, thereby balancing policy relevance and projection stability.

Analyses were conducted separately for each outcome and stratified by sex (male, female, and both sexes combined). In the BAPC framework, counts in each age-period cell were assumed to follow a Poisson distribution with the corresponding population denominator as an offset. Age, period, and cohort effects were modeled on the log scale, with second-order smoothness priors applied to improve stability of estimation and extrapolation [25]. Models were implemented in R (version 4.3.0) using a BAPC projection framework [26], and future population denominators were obtained from the corresponding IHME/GBD population projections [22]. From the fitted models, we derived annual ASIRs, ASPRs, and YLD rates for 2024–2040 using the same age-restricted GBD world standard population as in the main analyses. Projected uncertainty was summarized using 95% credible intervals (CrIs).

### 2.8. Statistical Analysis

All analyses were performed in R (version 4.3.0; R Foundation for Statistical Computing, Vienna, Austria), with the exception of Joinpoint regression, which was implemented using the Joinpoint Regression Program (version 5.0.2; Surveillance Research Program, National Cancer Institute, USA) [17]. Percentage changes between 1990 and 2023 were calculated from point estimates using the following formula:

$$\mathrm{Percentage}\;\mathrm{change}=\frac{{\mathrm{Estimate}}_{2023}-{\mathrm{Estimate}}_{1990}}{{\mathrm{Estimate}}_{1990}}\times 100\%$$

These percentage changes were not derived by comparing the overlap of UIs between the two years.

We distinguished three types of interval estimates: (i) 95% UIs for GBD estimates, derived from the 2.5th and 97.5th percentiles of the ordered draw-level distributions provided by GBD; (ii) 95% CIs for regression-based parameters, including APCs, AAPCs, EAPCs, net drifts, local drifts, and rate ratios; and (iii) 95% CrIs for BAPC projections, derived from the posterior distributions. Statistical significance was inferred when the corresponding interval did not include the null value (zero for absolute differences and percentage changes; one for rate ratios). Given the descriptive and hypothesis-generating nature of this study, no adjustment was made for multiple comparisons. Results are reported with descriptive precision to two decimal places unless otherwise specified.

## 3. Results

### 3.1. Overall Burden of Low Back Pain in China, 1990 and 2023

In 2023, population-level GBD estimates indicated that among Chinese adults aged 45 years and older, LBP accounted for 30.29 million incident cases (95% UI 21.24–41.26), 71.54 million prevalent cases (51.74–95.85), and 7.90 million YLDs (4.96–11.92). Compared with 1990, these numbers increased by 101.54%, 97.08%, and 96.11%, respectively, indicating that the absolute burden of LBP in this population had nearly doubled over the study period (Table 1).

In contrast, all three age-restricted age-standardized measures declined between 1990 and 2023. The truncated ASIR fell from 6259.65 (95% UI 4334.92–8558.89) to 4710.60 (3303.26–6409.34) per 100,000 (EAPC −0.53, 95% CI −0.61 to −0.46), the truncated ASPR from 15,188.97 to 11,109.73 per 100,000 (EAPC −0.58, 95% CI −0.66 to −0.49), and the truncated age-standardized YLD rate from 1672.96 to 1229.01 per 100,000 (EAPC −0.57, 95% CI −0.66 to −0.49) (Table 1).

Females consistently had higher age-standardized burdens than males. In 2023, female ASRs were 64% higher than male ASRs for incidence (5823.39 vs. 3554.94 per 100,000), 62% higher for prevalence (13,662.82 vs. 8429.31), and 61% higher for YLDs (1506.79 vs. 938.72). Sex differences in long-term declines were modest, with males showing a slightly faster decrease in incidence and females slightly faster decreases in prevalence and YLD rates.

### 3.2. Joinpoint Analysis of Temporal Trends in Low Back Pain, 1990–2023

**Figure 1:**
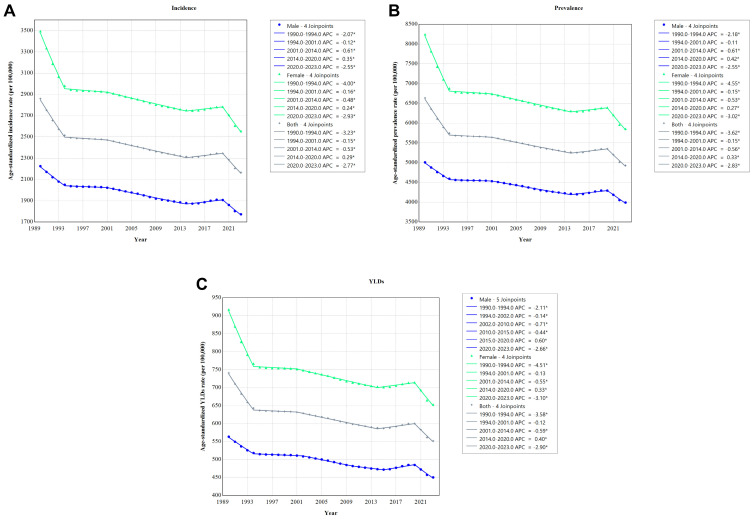
Temporal trends in age-restricted age-standardized incidence, prevalence, and YLD rates of low back pain among Chinese adults aged 45 years and older, by sex, 1990–2023. (**A**) Truncated age-standardized incidence rates; (**B**) truncated age-standardized prevalence rates; and (**C**) truncated age-standardized YLD rates. Solid lines represent annual modeled estimates, and fitted Joinpoint segments indicate changes in temporal trends. YLDs, years lived with disability.

Joinpoint regression based on annual modeled point estimates suggested that the decline in age-restricted age-standardized LBP burden was not linear over time but occurred in distinct phases (Table 2; Figure 1). For both sexes combined, joinpoint-derived AAPCs were −0.84 (95% CI −0.89 to −0.79) for incidence, −0.90 (−0.95 to −0.84) for prevalence, and −0.89 (−0.95 to −0.83) for YLDs, suggesting an overall long-term reduction in all three indicators.

Across all three outcomes, a common temporal pattern emerged for both sexes combined. ASRs declined steeply during 1990–1994 (APCs −3.23 to −3.62), were nearly stable during 1994–2001 (−0.12 to −0.15), and then resumed a moderate decline during 2001–2014 (−0.53 to −0.59). This was followed by a modest Joinpoint-identified increase during 2014–2020 (0.29 to 0.40), before a renewed decline during 2020–2023 (−2.77 to −2.90). Because uncertainty in the underlying GBD estimates was not propagated into the Joinpoint model, these short-term changes should be interpreted cautiously. The rebound in the late 2010s was seen consistently across incidence, prevalence, and YLDs, although its magnitude remained modest compared with the long-term downward trend.

Sex-specific patterns were broadly similar, but women experienced somewhat sharper changes at both ends of the period. Females showed steeper declines than males during 1990–1994 and again during 2020–2023, whereas the temporary increase during 2014–2020 was more pronounced in males, particularly for prevalence (APC 0.42 vs. 0.27 in females). These findings suggest that the overall downward trend in age-restricted age-standardized LBP burden concealed short-term reversals and sex-specific fluctuations that would not have been apparent from overall EAPCs alone. However, these short-term changes should be interpreted as exploratory patterns in modeled estimates.

### 3.3. Decomposition of Changes in Low Back Pain Burden, 1990–2023

**Figure 2:**
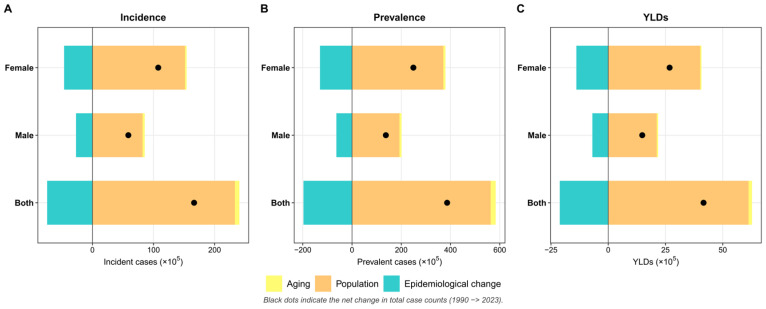
Decomposition of changes in low back pain burden among Chinese adults aged 45 years and older, 1990–2023. Changes in (**A**) incident cases, (**B**) prevalent cases, and (**C**) YLDs were decomposed into contributions from growth of the population aged 45 years and older, ageing within the ≥45 population, and epidemiological change, stratified by sex. Positive values indicate components contributing to increases in burden, whereas negative values indicate offsetting effects. YLDs, years lived with disability.

Decomposition analysis indicated that the increase in absolute LBP burden from 1990 to 2023 was driven overwhelmingly by demographic change, particularly population growth, while improvements in age-specific rates offset part of this increase (Figure 2; Supplementary Table S1). For both sexes combined, total counts increased by 16.64 million for incidence, 38.61 million for prevalence, and 4.16 million for YLDs. Growth of the population aged 45 years and older contributed 140.1% of the increase in incidence, 145.8% of the increase in prevalence, and 147.2% of the increase in YLDs, whereas ageing within the ≥45 population made much smaller positive contributions (4.4%, 5.3%, and 3.7%, respectively). By contrast, epidemiological change contributed negatively, offsetting 44.6% of the increase in incidence, 51.1% of the increase in prevalence, and 50.9% of the increase in YLDs.

The same broad pattern was observed in both sexes, but the offsetting effect of epidemiological change was generally stronger in females than in males. For prevalence, epidemiological change offset 52.2% of the increase in females compared with 46.4% in males; for YLDs, the corresponding offsets were 52.0% and 46.7%, respectively. Taken together, the decomposition shows that the historical expansion of LBP burden in middle-aged and older adults in China was primarily driven by expansion of the population aged 45 years and older, occurring in parallel with declining age-restricted age-standardized rates.

### 3.4. Age, Period, and Cohort Patterns of Low Back Pain Burden

**Figure 3:**
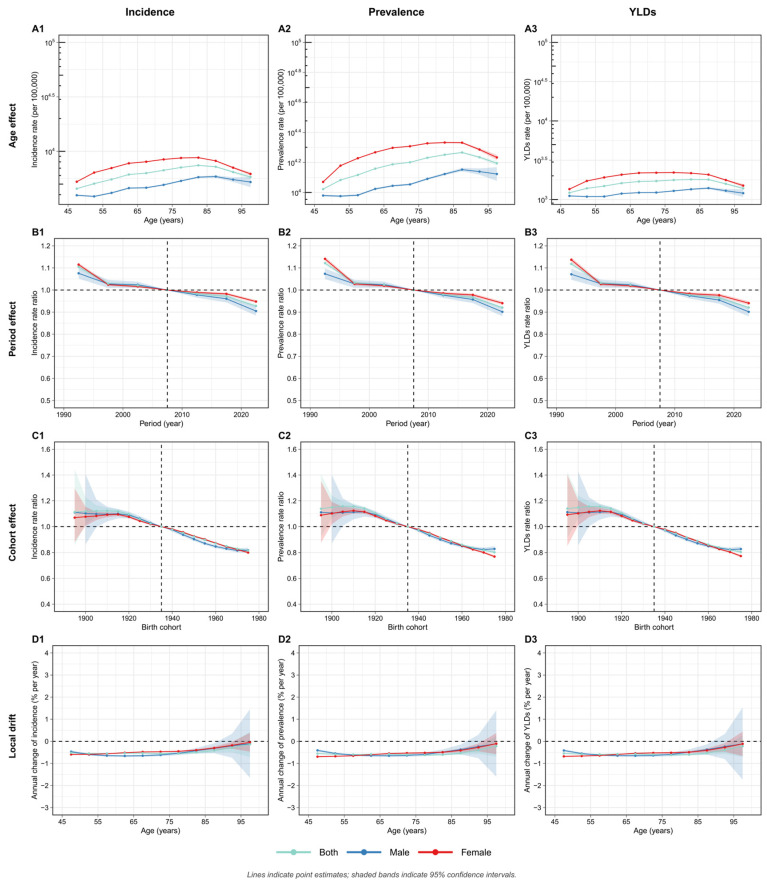
Descriptive age–period–cohort patterns of low back pain burden among Chinese adults aged 45 years and older, by sex. Panels (**A1**–**A3**) show longitudinal age curves, panels (**B1**–**B3**) period-related rate ratios, panels (**C1**–**C3**) cohort-related rate ratios, and panels (**D1**–**D3**) local drifts for incidence, prevalence, and YLDs, respectively. Shaded areas indicate 95% confidence intervals. Age–period–cohort estimates should be interpreted as descriptive estimable functions rather than separable causal age, period, and cohort effects. YLDs, years lived with disability.

Age–period–cohort analysis revealed descriptive age-, period-, and cohort-related patterns across incidence, prevalence, and YLDs (Figure 3; Supplementary Tables S2–S5). For both sexes combined, net drift indicated substantial cumulative declines over 1990–2023, amounting to 48.15% for incidence, 54.78% for prevalence, and 54.30% for YLDs (Supplementary Table S2). Net drift was similar between sexes for prevalence and YLDs, whereas the cumulative decline in incidence was somewhat greater in males than in females (49.87% vs. 42.58%).

Local drifts were negative across most age groups, indicating that age-specific rates generally declined over time (Figure 3(D1–D3); Supplementary Table S3). For incidence and prevalence, the most pronounced declines occurred in the middle-to-older age range, particularly around 60–74 years, whereas declines attenuated at more advanced ages. YLDs showed a similar pattern, with slower declines and wider confidence intervals in the oldest age groups, suggesting greater uncertainty and less marked improvement at extreme ages.

The longitudinal age curves showed that fitted age-specific rates rose with age, peaking at approximately 80–90 years for incidence and prevalence and at 65–75 years for YLDs, before plateauing or declining slightly at the oldest ages (Figure 3(A1–A3)). Across the age spectrum, females maintained higher fitted rates than males. Estimated period-related rate ratios declined progressively over calendar time (Figure 3(B1–B3); Supplementary Table S4). For both sexes combined, the prevalence period RR decreased from 1.122 (95% CI 1.113–1.131) in 1992.5 to 0.921 (0.913–0.928) in 2022.5 relative to the reference period (2007.5), with similar patterns for incidence and YLDs. Estimated cohort-related rate ratios also suggested lower risks among later-born generations (Figure 3(C1–C3); Supplementary Table S5): compared with the reference cohort born in 1935, earlier cohorts generally had higher risks, whereas later-born cohorts had progressively lower risks. For prevalence, the cohort RR declined to 0.802 (95% CI 0.793–0.811) in the 1975 cohort.

### 3.5. Burden Attributable to Modifiable Risk Factors

**Figure 4:**
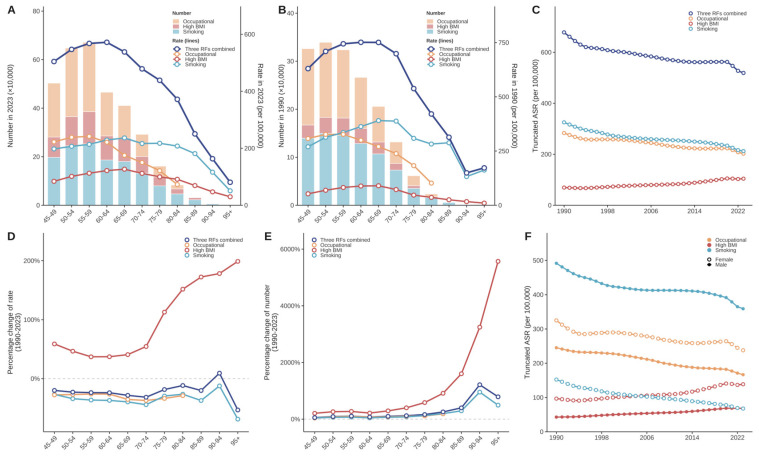
Low back pain YLDs attributable to three GBD-quantified risk factors among Chinese adults aged 45 years and older. (**A**) Age-specific attributable YLD numbers and corresponding truncated attributable age-standardized YLD rates in 2023; (**B**) age-specific attributable YLD numbers and corresponding truncated attributable age-standardized YLD rates in 1990; (**C**) annual truncated attributable age-standardized YLD rates for both sexes combined, 1990–2023; (**D**) percentage change in truncated attributable age-standardized YLD rates between 1990 and 2023 by age group; (**E**) percentage change in attributable YLD numbers between 1990 and 2023 by age group; and (**F**) annual truncated attributable age-standardized YLD rates by sex, 1990–2023. In panels (**A**,**B**), bars represent attributable YLD numbers and lines represent truncated attributable age-standardized YLD rates. Risk factors include high body mass index, occupational ergonomic factors, and smoking. YLDs, years lived with disability.

**Figure 5:**
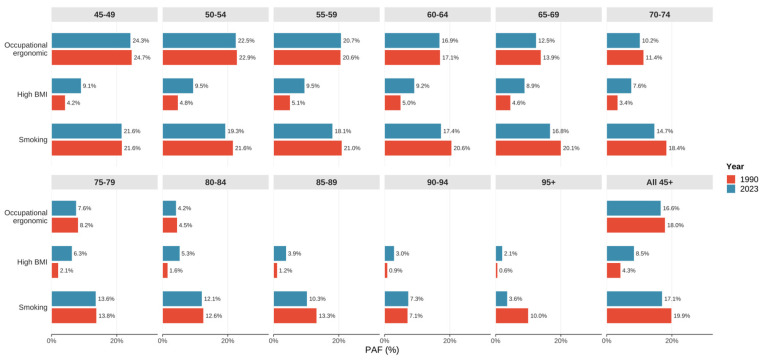
Age-specific population attributable fractions of low back pain due to three GBD-quantified risk factors among Chinese adults aged 45 years and older in 1990 and 2023. Population attributable fractions are shown for high body mass index, occupational ergonomic factors, and smoking across 5-year age groups. PAF, population attributable fraction.

The composition of LBP YLDs attributable to the three GBD-quantified risk factors shifted markedly between 1990 and 2023 (Figure 4 and Figure 5; Supplementary Table S6). In 1990, smoking accounted for the largest share of attributable YLDs among adults aged 45 years and older (47.56%), followed by occupational ergonomic factors (42.12%) and high BMI (10.32%). By 2023, the shares attributable to smoking and occupational ergonomic factors had both declined, to 41.19% and 38.37%, respectively, whereas the share attributable to high BMI increased to 20.43%, making it a substantially more prominent contributor than at baseline.

Attributable YLD numbers increased for all three risk factors, but their age-restricted age-standardized attributable YLD rate trajectories diverged. Attributable YLDs due to occupational ergonomic factors increased by 77.1%, from 709,738 in 1990 to 1,257,004 in 2023, while the corresponding truncated age-standardized rate declined (EAPC −0.81, 95% CI −0.87 to −0.75). Smoking-attributable YLDs increased by 68.3%, from 801,569 to 1,349,271, and showed the steepest decline in truncated age-standardized rate (EAPC −0.97, −1.05 to −0.88). In contrast, high body mass index showed the largest rise in attributable YLDs, increasing by 284.9% from 173,921 to 669,336, and was the only GBD-quantified risk factor with an increasing truncated attributable ASR (EAPC 1.43, 95% CI 1.33 to 1.53).

Sex-specific trends were not uniform. Declines in smoking-attributable rates were much steeper in females than in males (EAPC −2.07 vs. −0.64), whereas declines in occupational ergonomic factor-attributable rates were steeper in males than in females (−1.10 vs. −0.63). High BMI-attributable rates increased in both sexes, slightly faster in males (1.53 vs. 1.34). Age-specific PAFs further highlighted the expansion of high BMI as a contributor to LBP burden: its PAF approximately doubled across most age groups, increasing from 4.2% to 9.1% at ages 45–49, from 5.0% to 9.2% at ages 60–64, and from 0.6% to 2.1% at ages 95 years and older, whereas the PAFs for smoking and occupational ergonomic factors were generally stable or slightly lower in 2023 than in 1990 (Figure 5).

### 3.6. Projected Burden of Low Back Pain Through 2040

**Figure 6:**
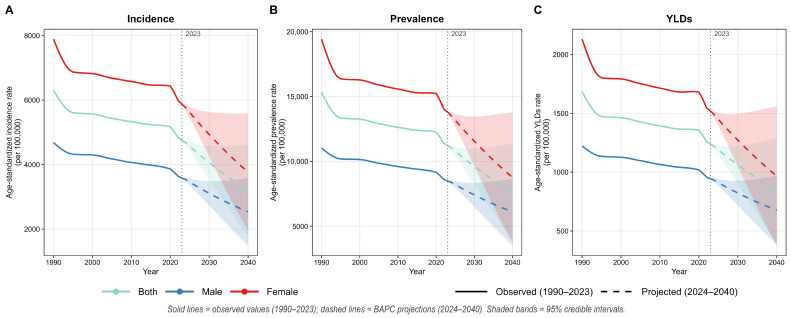
Observed and projected age-restricted age-standardized burden of low back pain among Chinese adults aged 45 years and older, by sex, 1990–2040. (**A**) Truncated age-standardized incidence rates, (**B**) truncated age-standardized prevalence rates, and (**C**) truncated age-standardized YLD rates. Solid lines indicate observed modeled estimates for 1990–2023, dashed lines indicate model-based projected estimates for 2024–2040, and shaded bands represent 95% credible intervals. YLDs, years lived with disability.

BAPC projections suggested that age-restricted age-standardized LBP burden may continue to decline through 2040 among Chinese adults aged 45 years and older, under the assumption that historical age–period–cohort patterns persist (Figure 6; Supplementary Table S7). Because formal back-validation was not performed, these projections should be interpreted as exploratory model-based estimates rather than definitive forecasts. For both sexes combined, using the model-fitted 2023 value as the projection baseline, the truncated ASIR was projected to decline from 4751.08 per 100,000 to 3197.07 per 100,000 in 2040, the truncated ASPR from 11,215.80 to 7620.89 per 100,000, and the truncated age-standardized YLD rate from 1238.05 to 843.90 per 100,000. The projected reductions are gradual in the near term and become larger by the mid-2030s, although uncertainty widens over the forecast horizon.

The same downward pattern is projected for both sexes, but females are expected to retain a substantially higher burden throughout the projection period. By 2040, the projected ASIR is 3766.30 per 100,000 in females and 2537.12 in males; the corresponding ASPRs are 8763.44 and 6105.88, and the YLD rates are 967.14 and 676.73, respectively. The widening 95% CrIs in later years indicate increasing uncertainty with longer projection horizons, but the direction of change remains consistently downward across all outcomes and both sexes.

## 4. Discussion

LBP among Chinese adults aged 45 years and older has undergone a marked epidemiological transition over the past three decades. Although truncated ASIRs, truncated ASPRs, and truncated age-standardized YLD rates declined between 1990 and 2023, the absolute numbers of incident cases, prevalent cases, and YLDs nearly doubled during the same period. This divergence between declining age-restricted standardized rates and increasing case counts was one of the central findings of our study and reflects the growing influence of demographic expansion of the middle-aged and older population on non-fatal health conditions. In practical terms, the increasing numbers of cases and YLDs indicate growing needs for pain management, rehabilitation, and functional support, whereas the declining truncated ASRs suggest improvement in the age-adjusted rate of LBP burden within the population aged 45 years and older. Females consistently experienced a higher burden than males, the attributable risk profile shifted toward a greater contribution from high BMI, and age-standardized burden is projected to continue declining through 2040. For an ageing society, the key challenge is therefore not simply whether age-restricted standardized rates fall, but whether health and social care systems can respond to the growing absolute number of middle-aged and older adults living with pain-related disability. This distinction is important because declining truncated ASRs should not be interpreted as a reduction in total service demand.

Our findings are broadly consistent with previous GBD-based studies showing that LBP remains one of the leading causes of non-fatal health loss globally and in China [1,6]. However, the present analysis extends previous work in several ways. First, we focused specifically on Chinese adults aged 45 years and older, rather than all-age populations, and recalculated age-restricted age-standardized rates for this policy-relevant group. Second, we distinguished the contributions of expansion of the population aged 45 years and older, ageing within this age range, and changes in age-specific epidemiology. Third, we jointly examined descriptive age–period–cohort patterns, GBD-defined risk-attributable YLDs, and future projections. This integrated approach provides evidence more directly relevant to healthy-ageing policy, rehabilitation planning, and functional disability prevention among middle-aged and older adults.

A central implication of our findings is that improvements in standardized epidemiological indicators do not necessarily translate into a reduced burden for older populations. Decomposition analysis showed that favorable changes in age-specific epidemiology contributed to lowering the burden of LBP, but these gains were outweighed by expansion of the population aged 45 years and older. In a country as populous and rapidly ageing as China [2,22], even modest declines in ASRs can coexist with substantial increases in the number of people affected by LBP. This distinction is particularly relevant in gerontology and geriatric care, because LBP in later life is closely linked to mobility limitation, reduced independence, sleep disturbance, frailty, and lower quality of life [27,28]. Therefore, declining relative rates should not be interpreted as a reduced need for clinical care, rehabilitation, or community-based disability prevention [7,8,9]. Rather, the burden of LBP in older Chinese adults is increasingly shaped by the interaction between epidemiological improvement and demographic ageing.

Sex differences were a persistent feature of the LBP burden. The higher burden observed among females is consistent with previous studies showing that LBP is more common and more disabling in women than in men [1,6,29]. Several mechanisms may contribute to this pattern. Biological factors, including sex-related differences in pain sensitivity, pain inhibition, hormonal changes, and musculoskeletal ageing, may increase vulnerability among women [30]. Socially patterned exposures may also be important, including unpaid caregiving, domestic labor, and gender differences in healthcare-seeking behavior [29,31]. In our study, females had higher ASIRs, ASPRs, and age-standardized YLD rates throughout the historical period and were projected to retain a higher burden through 2040. Nevertheless, some temporal indicators suggested slightly faster declines among females in prevalence and YLDs, implying that the sex gap may narrow modestly over time, although it is unlikely to disappear. These findings support the need for sex-sensitive prevention and rehabilitation strategies, especially for older women who may experience both biological vulnerability and accumulated social disadvantage.

The age–period–cohort model results further emphasize the importance of a life-course perspective, while remaining descriptive and hypothesis-generating rather than causal. Longitudinal age curves showed that LBP burden increased substantially with age, and local drifts indicated that rate declines were least pronounced in the oldest age groups. This suggests that recent improvements have not been distributed evenly across later life, with the oldest adults showing less pronounced declines than younger-old adults. Such a pattern is consistent with evidence that functional vulnerability and disability accumulate sharply at advanced ages in China [32]. Later calendar periods and later-born cohorts were associated with lower estimated rate ratios in descriptive APC contrasts. However, these patterns should not be interpreted as uniquely identifiable causal period or cohort effects because the linear components of age, period, and cohort cannot be separated without modeling constraints. They are nevertheless compatible with broader social and health transitions across successive generations, including changes in living conditions, education, occupational structure, healthcare access, and health awareness [33,34]. The persistence of high fitted rates in older age groups indicates that these broader improvements have not eliminated the disabling impact of LBP in late life [7,9].

Risk-attribution findings indicate that the structure of LBP burden attributable to GBD-quantified modifiable risk factors has changed in important ways. Smoking and occupational ergonomic factors remained major contributors to attributable YLDs in 2023, but both showed declining shares and declining age-restricted age-standardized attributable rates over time. Smoking was included because it is treated as a risk-outcome pair for LBP in the GBD CRA framework, based on epidemiological evidence linking smoking to LBP through mechanisms such as impaired intervertebral disc nutrition, vascular and inflammatory pathways, and pain-related processes. Nevertheless, the CRA estimates should be interpreted as population-level attributable burden rather than as evidence of individual-level causality. In contrast, high BMI showed the largest increase in attributable YLD numbers, was the only risk factor with rising age-standardized attributable rates, and had higher population attributable fractions across nearly all age groups. This pattern is consistent with growing evidence that high BMI is becoming an increasingly important driver of LBP burden both globally and in China [1,3,5]. For middle-aged and older adults, excess body weight may increase spinal loading and interact with broader metabolic and functional vulnerability [35]. These findings suggest that traditional strategies, such as tobacco control and ergonomic protection, remain necessary but are unlikely to be sufficient alone. Weight management, maintenance of muscle strength, physical activity promotion, and broader metabolic health interventions may become increasingly important components of LBP prevention in ageing populations [3,7].

Projection results suggest that age-restricted age-standardized LBP burden may continue to decline through 2040, indicating possible further epidemiological improvement under the assumption that historical age–period–cohort patterns persist. However, this finding should be interpreted cautiously because the projections are model-based, were not formally back-validated in this analysis, and do not account for unforeseen changes in obesity, occupational exposure, healthcare access, clinical practice, or health policy. Projected declines begin from a high baseline burden, females are expected to maintain substantially higher rates than males, and historical analyses already show that falling standardized rates can coexist with persistently large service needs in ageing populations [2,7,8,9,22]. For China, where population ageing is progressing rapidly, the absolute number of older adults requiring pain management, rehabilitation, mobility support, and long-term functional care may remain substantial. Future planning should therefore consider both standardized rates and absolute burden. Strengthening musculoskeletal care, integrating LBP management into geriatric assessment, expanding community-based rehabilitation, and promoting disability prevention may be particularly important within the broader framework of healthy ageing and Healthy China 2030 [12].

A major strength of this study is its focus on a policy-relevant population spanning late working life and older adulthood, a period in which LBP contributes to both productivity loss and later-life functional decline. By integrating decomposition analysis, age–period–cohort modeling, risk attribution, and future projection within a consistent GBD framework, this study provides a comprehensive picture of LBP burden among Chinese adults aged 45 years and older. This approach also allowed us to distinguish between increasing absolute burden and declining age-restricted standardized rates, an important distinction for health-service planning in an ageing population.

Findings should nevertheless be interpreted in light of several limitations. All estimates were derived from GBD 2023 modeled outputs rather than from a single directly observed national surveillance system, and therefore depend on the availability and representativeness of underlying data sources as well as on GBD modeling assumptions, cross-walk adjustments, interpolation, and extrapolation. Because aggregated age-sex-year estimates were used, the results should be interpreted at the population level rather than as evidence of individual-level causal relationships. National-level analyses could not capture provincial, urban–rural, or socioeconomic differences in LBP burden, risk-factor exposure, healthcare access, rehabilitation resources, or demographic ageing within China. Temporal patterns may also have been influenced by changes in healthcare-seeking behavior, diagnostic practices, survey coverage, reporting, and access to rehabilitation services. In addition, Joinpoint and EAPC analyses were based on annual modeled point estimates without propagation of the full GBD uncertainty into the trend models; short-term changes should therefore be viewed as descriptive patterns rather than definitive evidence of abrupt epidemiological shifts. The recalculated age-restricted age-standardized rates, or truncated ASRs, are useful for comparisons within adults aged 45 years and older but are not directly comparable with standard all-age GBD ASRs. Risk-attribution estimates were limited to the risk factors quantified for LBP in the GBD CRA framework and should be interpreted as population-level attributable burden rather than individual-level causal effects. Similarly, age–period–cohort results describe estimable age-, period-, and cohort-related patterns rather than separable causal effects. Finally, BAPC projections were not formally back-validated or compared with alternative projection models and should be viewed as exploratory model-based estimates. Because the 2020–2023 period coincided with the COVID-19 era, during which healthcare utilization, physical activity, occupational exposure, rehabilitation access, and pain reporting may have changed, short-term trends during this period and projections based partly on these recent data should be interpreted cautiously.

Taken together, these findings suggest that the future burden of LBP among middle-aged and older adults in China will be shaped by the balance between epidemiological improvement, demographic ageing, and changing risk-factor profiles. The burden is shifting rather than simply diminishing. For an ageing society, this means that continued reductions in standardized rates must be accompanied by strategies that preserve function, reduce disability, and support independence in later life. Effective responses will require not only prevention and treatment of LBP, but also adaptation of geriatric and rehabilitation services to a population in which the absolute number of people living with LBP-related disability remains large.

## 5. Conclusions

Low back pain remains a substantial and evolving challenge to healthy ageing in China. Among adults aged 45 years and older, absolute LBP burden increased markedly from 1990 to 2023 despite declining age-restricted age-standardized rates, and high body mass index became increasingly important as a contributor to attributable disability. In an ageing population, effective responses should combine risk-factor control with rehabilitation, functional support, and age-sensitive care to reduce disability and maintain independence in later life.

## Figures and Tables

**Table 1:** Low back pain burden among Chinese adults aged 45 years and older by sex in 1990 and 2023, with estimated annual percentage changes in age-restricted age-standardized rates.

Measure	Sex	1990_Millions (95% UI)	2023_Millions (95% UI)	Percentage Change (%)	1990 Age-Restricted ASR per 100,000 (95% UI)	2023 Age-Restricted ASR per 100,000 (95% UI)	EAPC in Age-Restricted ASR (95% CI)
Incidence	Both	15.03 (10.39, 20.56)	30.29 (21.24, 41.26)	101.54	6259.65 (4334.92, 8558.89)	4710.60 (3303.26, 6409.34)	−0.53 (−0.61, −0.46)
	Male	5.58 (3.81, 7.65)	11.07 (7.65, 15.17)	98.48	4647.63 (3181.80, 6380.00)	3554.94 (2461.11, 4868.70)	−0.58 (−0.63, −0.52)
	Female	9.46 (6.55, 12.91)	19.23 (13.52, 26.19)	103.34	7835.56 (5434.17, 10,691.89)	5823.39 (4095.91, 7926.47)	−0.51 (−0.60, −0.42)
Prevalence	Both	36.30 (25.97, 48.99)	71.54 (51.74, 95.85)	97.08	15,188.97 (10,881.33, 20,482.87)	11,109.73 (8039.61, 14,896.00)	−0.58 (−0.66, −0.49)
	Male	13.12 (9.29, 17.91)	26.18 (18.86, 35.45)	99.59	10,954.89 (7762.88, 14,941.26)	8429.31 (6076.47, 11,407.27)	−0.56 (−0.62, −0.51)
	Female	23.18 (16.60, 31.14)	45.36 (32.77, 60.74)	95.66	19,265.22 (13,802.01, 25,876.98)	13,662.82 (9871.48, 18,320.73)	−0.59 (−0.70, −0.48)
YLDs	Both	4.03 (2.51, 6.06)	7.90 (4.96, 11.92)	96.11	1672.96 (1046.54, 2511.51)	1229.01 (769.29, 1852.83)	−0.57 (−0.66, −0.49)
	Male	1.47 (0.91, 2.24)	2.92 (1.81, 4.41)	98.17	1216.34 (753.07, 1848.22)	938.72 (581.55, 1417.96)	−0.57 (−0.62, −0.51)
	Female	2.56 (1.60, 3.83)	4.99 (3.15, 7.50)	94.93	2116.92 (1326.72, 3171.09)	1506.79 (947.93, 2269.36)	−0.59 (−0.69, −0.48)

Abbreviations: YLDs, years lived with disability; ASR, age-standardized rate; truncated ASR, age-restricted age-standardized rate; EAPC, estimated annual percentage change; UI, uncertainty interval; CI, confidence interval. Note: Truncated ASRs were calculated by direct standardization using the GBD world standard population restricted to age groups 45–49 through 95+ years, with weights renormalized within this age range. These rates are not directly comparable with standard all-age GBD ASRs. Percentage changes were calculated from point estimates. EAPCs were estimated from annual truncated ASRs using simple log-linear regression and should be interpreted as supplementary overall trend summaries, not as equivalent to Joinpoint-derived AAPCs or endpoint-based annualized changes.

**Table 2:** Joinpoint regression analysis of truncated age-standardized low back pain rates among Chinese adults aged 45 years and older, 1990–2023.

		Segment 1		Segment 2		Segment 3		Segment 4		Segment 5		Overall
Measure	Sex	Period	APC (95% CI)	Period	APC (95% CI)	Period	APC (95% CI)	Period	APC (95% CI)	Period	APC (95% CI)	AAPC (95% CI)
Incidence	Both	1990–1994	−3.23 (−3.44, −3.02)	1994–2001	−0.15 (−0.26, −0.04)	2001–2014	−0.53 (−0.57, −0.49)	2014–2020	0.29 (0.15, 0.44)	2020–2023	−2.77 (−3.08, −2.46)	−0.84 (−0.89, −0.79)
	Male	1990–1994	−2.07 (−2.27, −1.87)	1994–2001	−0.12 (−0.23, −0.01)	2001–2014	−0.61 (−0.65, −0.57)	2014–2020	0.35 (0.21, 0.49)	2020–2023	−2.55 (−2.85, −2.26)	−0.69 (−0.74, −0.64)
	Female	1990–1994	−4.00 (−4.21, −3.79)	1994–2001	−0.16 (−0.28, −0.05)	2001–2014	−0.48 (−0.52, −0.44)	2014–2020	0.24 (0.09, 0.39)	2020–2023	−2.93 (−3.25, −2.61)	−0.94 (−0.99, −0.89)
Prevalence	Both	1990–1994	−3.62 (−3.85, −3.40)	1994–2001	−0.15 (−0.27, −0.02)	2001–2014	−0.56 (−0.61, −0.52)	2014–2020	0.33 (0.17, 0.50)	2020–2023	−2.83 (−3.18, −2.48)	−0.90 (−0.95, −0.84)
	Male	1990–1994	−2.18 (−2.39, −1.97)	1994–2001	−0.11 (−0.22, 0.00)	2001–2014	−0.61 (−0.65, −0.57)	2014–2020	0.42 (0.26, 0.57)	2020–2023	−2.55 (−2.87, −2.22)	−0.69 (−0.74, −0.64)
	Female	1990–1994	−4.55 (−4.78, −4.31)	1994–2001	−0.15 (−0.28, −0.02)	2001–2014	−0.53 (−0.58, −0.49)	2014–2020	0.27 (0.10, 0.44)	2020–2023	−3.02 (−3.39, −2.64)	−1.03 (−1.09, −0.97)
YLDs	Both	1990–1994	−3.58 (−3.80, −3.35)	1994–2001	−0.12 (−0.25, 0.00)	2001–2014	−0.59 (−0.63, −0.54)	2014–2020	0.40 (0.24, 0.57)	2020–2023	−2.90 (−3.26, −2.54)	−0.89 (−0.95, −0.83)
	Male	1990–1994	−2.10 (−2.32, −1.88)	1994–2002	−0.16 (−0.25, −0.06)	2002–2014	−0.66 (−0.71, −0.61)	2014–2020	0.51 (0.35, 0.67)	2020–2023	−2.60 (−2.95, −2.26)	−0.68 (−0.74, −0.63)
	Female	1990–1994	−4.51 (−4.75, −4.28)	1994–2001	−0.13 (−0.26, 0.01)	2001–2014	−0.55 (−0.60, −0.51)	2014–2020	0.33 (0.16, 0.51)	2020–2023	−3.10 (−3.47, −2.73)	−1.03 (−1.09, −0.97)

Abbreviations: APC, annual percentage change; AAPC, average annual percentage change; CI, confidence interval; YLDs, years lived with disability. Note: Joinpoint analyses were based on annual modeled point estimates of truncated age-standardized rates. Because uncertainty in the underlying GBD estimates was not propagated into the models, APCs and AAPCs should be interpreted as descriptive trend summaries. AAPCs summarize segmented Joinpoint trends and are not directly equivalent to EAPCs from simple log-linear regression.

## Data Availability

The data used in this study are publicly available from the Global Burden of Disease Study 2023 Results Tool, provided by the Institute for Health Metrics and Evaluation (IHME), at https://vizhub.healthdata.org/gbd-results/ (accessed on 10 April 2026). All data analyzed in this study were obtained from this publicly accessible source.

## Supplementary Materials

The following supporting information can be downloaded at: https://www.mdpi.com/article/10.3390/healthcare14121692/s1, Tables S1–S7: Table S1. Decomposition of changes in incident cases, prevalent cases, and YLDs of low back pain among Chinese adults aged 45 years and older, 1990–2023. Table S2. Cumulative declines estimated from net drift in age-period-cohort analysis of incidence, prevalence, and YLDs of low back pain among Chinese adults aged 45 years and older, 1990–2023. Table S3. Local drift estimates from age-period-cohort analysis of low back pain incidence, prevalence, and YLDs among Chinese adults aged 45 years and older. Table S4. Period rate ratios from age-period-cohort analysis of low back pain incidence, prevalence, and YLDs among Chinese adults aged 45 years and older. Table S5. Cohort rate ratios from age-period-cohort analysis of low back pain incidence, prevalence, and YLDs among Chinese adults aged 45 years and older. Table S6. Attributable YLD numbers, truncated age-standardized attributable YLD rates, and population attributable fractions for major modifiable risk factors of low back pain among Chinese adults aged 45 years and older, 1990–2023. Table S7. Observed and projected age-standardized incidence, prevalence, and YLD rates of low back pain among Chinese adults aged 45 years and older, by sex, 1990–2040.

## Abbreviations

AAPC, average annual percentage change; APC, annual percentage change; ASR, age-standardized rate; ASIR, age-standardized incidence rate; ASPR, age-standardized prevalence rate; BAPC, Bayesian age–period–cohort; BMI, body mass index; CI, confidence interval; CrI, credible interval; CRA, comparative risk assessment; EAPC, estimated annual percentage change; GBD, Global Burden of Disease; IHME, Institute for Health Metrics and Evaluation; LBP, low back pain; PAF, population attributable fraction; RR, rate ratio; UI, uncertainty interval; YLDs, years lived with disability.

## References

[1] Ferreira M.L., De Luca K., Haile L.M., Steinmetz J.D., Culbreth G.T., Cross M., Kopec J.A., Ferreira P.H., Blyth F.M., Buchbinder R. (2023). Global, regional, and national burden of low back pain, 1990–2020, its attributable risk factors, and projections to 2050: A systematic analysis of the Global Burden of Disease Study 2021. Lancet Rheumatol..

[2] National Bureau of Statistics of China (2024). Statistical Communiqué of the People’s Republic of China on the 2023 National Economic and Social Development.

[3] Xu J., Li J., Huang H., Lin T., Liao Z., Zhang W., Wu J., Wu F. (2025). High-BMI-related low back pain in China: A GBD-based observational study on sex-age trends and projections (1990–2021). Eur. J. Med. Res..

[4] Gu H., Hou J., Fu X., You J. (2026). Associations of physical activity and sedentary behavior with chronic low back pain in middle-aged and older adults: A cross-sectional study. Front. Public Health.

[5] Li Q., Peng L., Wang Y., Yang Y., Wang Z. (2024). Risk factors for low back pain in the Chinese population: A systematic review and meta-analysis. BMC Public Health.

[6] Wu A., Dong W., Liu S., Cheung J.P.Y., Kwan K.Y.H., Zeng X., Zhang K., Sun Z., Wang X., Cheung K.M.C. (2019). The prevalence and years lived with disability caused by low back pain in China, 1990 to 2016: Findings from the global burden of disease study 2016. Pain.

[7] Foster N.E., Anema J.R., Cherkin D., Chou R., Cohen S.P., Gross D.P., Ferreira P.H., Fritz J.M., Koes B.W., Peul W. (2018). Prevention and treatment of low back pain: Evidence, challenges, and promising directions. Lancet.

[8] Schofield D., Kelly S., Shrestha R., Callander E., Passey M., Percival R. (2012). The impact of back problems on retirement wealth. Pain.

[9] Hartvigsen J., Hancock M.J., Kongsted A., Louw Q., Ferreira M.L., Genevay S., Hoy D., Karppinen J., Pransky G., Sieper J. (2018). What low back pain is and why we need to pay attention. Lancet.

[10] Jiang X., Wang R., Bai Y.-W., Tang L., Xing W.-Y., Chen N., Wang X.-Q. (2025). Prevalence and risk factors of low back pain in middle-aged and older adult in China: A cross-sectional study. Arch. Public Health.

[11] Yao W., Mai X., Luo C., Ai F., Chen Q. (2011). A cross-sectional survey of nonspecific low back pain among 2083 schoolchildren in China. Spine.

[12] Tan X., Liu X., Shao H. (2017). Healthy China 2030: A vision for health care. Value Health Reg. Issues.

[13] Global Burden of Disease Collaborative Network (2025). Global Burden of Disease Study 2023 (GBD 2023) Results.

[14] Hay S.I., Ong K.L., Santomauro D.F., Aalipour M.A., Aalruz H., Ababneh H.S., Abaraogu U.O., Abate B.B., Abbafati C., Abbas N. (2025). Burden of 375 diseases and injuries, risk-attributable burden of 88 risk factors, and healthy life expectancy in 204 countries and territories, including 660 subnational locations, 1990–2023: A systematic analysis for the Global Burden of Disease Study 2023. Lancet.

[15] Zhang C., Lv B., Yi Q., Qiu G., Wu F. (2025). Global, regional, and national burden of low back pain in working-age population from 1990 to 2021 and projections for 2050. Front Public Health.

[16] Salomon J.A., Haagsma J.A., Davis A., De Noordhout C.M., Polinder S., Havelaar A.H., Cassini A., Devleesschauwer B., Kretzschmar M., Speybroeck N. (2015). Disability weights for the Global Burden of Disease 2013 study. Lancet Glob. Health.

[17] National Cancer Institute (2023). Joinpoint Regression Program.

[18] Kim H.J., Fay M.P., Feuer E.J., Midthune D.N. (2000). Permutation tests for joinpoint regression with applications to cancer rates. Stat. Med..

[19] National Cancer Institute Joinpoint Regression Program Technical Help. https://surveillance.cancer.gov/joinpoint.

[20] Clegg L.X., Hankey B.F., Tiwari R., Feuer E.J., Edwards B.K. (2009). Estimating average annual per cent change in trend analysis. Stat. Med..

[21] Gupta P.D. (1993). Standardization and Decomposition of Rates: A User’s Manual.

[22] GBD 2023 Demographics Collaborators (2025). Global age-sex-specific all-cause mortality and life expectancy estimates for 204 countries and territories and 660 subnational locations, 1950–2023: A demographic analysis for the Global Burden of Disease Study 2023. Lancet.

[23] Holford T.R. (1991). Understanding the effects of age, period, and cohort on incidence and mortality rates. Annu. Rev. Public Health.

[24] Rosenberg P.S., Check D.P., Anderson W.F. (2014). A web tool for age–period–cohort analysis of cancer incidence and mortality rates. Cancer Epidemiol. Biomark. Prev..

[25] Fosse E. (2020). Bayesian age–period–cohort models. Age, Period and Cohort Effects.

[26] Riebler A., Held L. (2017). Projecting the future burden of cancer: Bayesian age–period–cohort analysis with integrated nested Laplace approximations. Biom. J..

[27] Docking R.E., Fleming J., Brayne C., Zhao J., Macfarlane G.J., Jones G.T. (2011). Epidemiology of back pain in older adults: Prevalence and risk factors for back pain onset. Rheumatology.

[28] Larsson C., Hansson E.E., Sundquist K., Jakobsson U. (2017). Chronic pain in older adults: Prevalence, incidence, and risk factors. Scand. J. Rheumatol..

[29] Bertakis K.D., Azari R., Helms L.J., Callahan E.J., Robbins J.A. (2000). Gender differences in the utilization of health care services. J. Fam. Pract..

[30] Mogil J.S. (2012). Sex differences in pain and pain inhibition: Multiple explanations of a controversial phenomenon. Nat. Rev. Neurosci..

[31] Chen L., Fan H., Chu L. (2020). The double-burden effect: Does the combination of informal care and work cause adverse health outcomes among females in China?. J. Aging Health.

[32] Zeng Y., Feng Q., Hesketh T., Christensen K., Vaupel J.W. (2017). Survival, disabilities in activities of daily living, and physical and cognitive functioning among the oldest-old in China: A cohort study. Lancet.

[33] Jiang J., Zhang X. (2020). Social transition and health inequality in China: An age-period-cohort analysis. Public Health.

[34] Zhou M., Wang H., Zhu J., Chen W., Wang L., Liu S., Li Y., Wang L., Liu Y., Yin P. (2016). Cause-specific mortality for 240 causes in China during 1990–2013: A systematic subnational analysis for the Global Burden of Disease Study 2013. Lancet.

[35] Shiri R., Karppinen J., Leino-Arjas P., Solovieva S., Viikari-Juntura E. (2010). The association between obesity and low back pain: A meta-analysis. Am. J. Epidemiol..

