# Supplementary material for: Low Back Pain in Chinese Adults Aged 45 Years and Older: Trends, Drivers, and Projections, 1990–2040
Source: Healthcare (Basel). 2026 Jun 12;14(12):1692. doi: 10.3390/healthcare14121692 (PMC13300509; doi:10.3390/healthcare14121692)
Supplement: Supplementary file 1 [file healthcare-14-01692-s001.zip › healthcare-4343988-supplementary.pdf]

**Supplementary Materials**	**Page**
**Supplementary Table S1. Decomposition of changes in incident cases, prevalent cases, and YLDs of low back pain among Chinese adults aged 45 years and older, 1990–2023**	2
**Supplementary Table S2. Cumulative declines estimated from net drift in age-period-cohort analysis of incidence, prevalence, and YLDs of low back pain among Chinese adults aged 45 years and older, 1990–2023**	3
**Supplementary Table S3. Local drift estimates from age-period-cohort analysis of low back pain incidence, prevalence, and YLDs among Chinese adults aged 45 years and older**	4-7
**Supplementary Table S4. Period rate ratios from age-period-cohort analysis of low back pain incidence, prevalence, and YLDs among Chinese adults aged 45 years and older**	8-10
**Supplementary Table S5. Cohort rate ratios from age-period-cohort analysis of low back pain incidence, prevalence, and YLDs among Chinese adults aged 45 years and older**	11-12
**Supplementary Table S6. Attributable YLD numbers, truncated age-standardized attributable YLD rates, and population attributable fractions for major modifiable risk factors of low back pain among Chinese adults aged 45 years and older, 1990–2023**	13
**Supplementary Table S7. Observed and projected age-standardized incidence, prevalence, and YLD rates of low back pain among Chinese adults aged 45 years and older, by sex, 1990–2040**	14-15

Supplementary Table S1. Decomposition of changes in incident cases, prevalent cases, and YLDs of low back pain among Chinese adults aged 45 years and older, 1990–2023

Measure	Sex	Total Change (millions)	Population Growth (millions)	Population Aging (millions)	Epidemiological Change (millions)
Incidence	Both	16.64	23.32 (140.1%)	0.74 (4.4%)	-7.42 (-44.6%)
	Male	5.88	8.20 (139.6%)	0.36 (6.2%)	-2.69 (-45.8%)
	Female	10.77	15.14 (140.6%)	0.27 (2.5%)	-4.64 (-43.1%)
Prevalence	Both	38.61	56.31 (145.8%)	2.04 (5.3%)	-19.73 (-51.1%)
	Male	13.73	19.27 (140.3%)	0.83 (6.0%)	-6.37 (-46.4%)
	Female	24.91	37.07 (148.8%)	0.85 (3.4%)	-13.01 (-52.2%)
YLDs	Both	4.16	6.13 (147.2%)	0.15 (3.7%)	-2.12 (-50.9%)
	Male	1.49	2.12 (142.5%)	0.06 (4.3%)	-0.69(-46.7%)
	Female	2.68	4.02 (149.8%)	0.06 (2.1%)	-1.39 (-52.0%)

Note: Decomposition analysis was performed using the Das Gupta method. Values in parentheses represent the percentage contribution of each component to the total change. Population growth refers to changes due to population size increase; population aging refers to changes due to shifts in age structure; epidemiological change refers to changes in age-specific rates.

Supplementary Table S2. Cumulative declines estimated from net drift in age-period-cohort analysis of incidence, prevalence, and YLDs of low back pain among Chinese adults aged 45 years and older, 1990–2023

Measure	Sex	Net Drift
Incidence	Both	-48.15 (-53.22, -43.07)
	Male	-49.87 (-63.05, -36.67)
	Female	-42.58 (-46.35, -38.80)
Prevalence	Both	-54.78 (-58.91, -50.65)
	Male	-50.92 (-63.73, -38.09)
	Female	-50.82 (-55.13, -46.52)
YLDs	Both	-54.30 (-58.58, -50.02)
	Male	-51.07 (-64.86, -37.27)
	Female	-50.21 (-55.07, -45.35)

Abbreviations: CI, Confidence Intervals. Net drift represents the overall annual percentage change in age-standardized rates after adjusting for age and period effects.

Supplementary Table S3. Local drift estimates from age-period-cohort analysis of low back pain incidence, prevalence, and YLDs among Chinese adults aged 45 years and older

Measure	Sex	Age Group	Local Drift (% per year)
Incidence	Both	47.5	-0.52 (-0.55, -0.49)
		52.5	-0.55 (-0.57, -0.53)
		57.5	-0.55 (-0.57, -0.53)
		62.5	-0.54 (-0.56, -0.52)
		67.5	-0.53 (-0.56, -0.51)
		72.5	-0.55 (-0.58, -0.52)
		77.5	-0.55 (-0.59, -0.51)
		82.5	-0.50 (-0.56, -0.45)
		87.5	-0.42 (-0.51, -0.32)
		92.5	-0.29 (-0.50, -0.08)
		97.5	-0.14 (-0.72, 0.44)
	Male	47.5	-0.47 (-0.53, -0.41)
		52.5	-0.60 (-0.64, -0.55)
		57.5	-0.65 (-0.69, -0.60)
		62.5	-0.66 (-0.71, -0.62)
		67.5	-0.65 (-0.70, -0.60)
		72.5	-0.62 (-0.68, -0.55)
		77.5	-0.54 (-0.62, -0.46)
		82.5	-0.43 (-0.55, -0.30)
		87.5	-0.32 (-0.54, -0.09)
		92.5	-0.20 (-0.75, 0.34)
		97.5	-0.11 (-1.65, 1.45)
	Female	47.5	-0.59 (-0.62, -0.57)
		52.5	-0.59 (-0.61, -0.57)
		57.5	-0.56 (-0.58, -0.54)
		62.5	-0.51 (-0.53, -0.50)
		67.5	-0.48 (-0.50, -0.46)
		72.5	-0.47 (-0.50, -0.45)
		77.5	-0.45 (-0.48, -0.42)
		82.5	-0.40 (-0.44, -0.36)
		87.5	-0.30 (-0.37, -0.23)

Measure	Sex	Age Group	Local Drift (% per year)
		92.5	-0.18 (-0.33, -0.02)
		97.5	-0.04 (-0.47, 0.38)
Prevalence	Both	47.5	-0.55 (-0.58, -0.53)
		52.5	-0.59 (-0.61, -0.57)
		57.5	-0.60 (-0.61, -0.58)
		62.5	-0.58 (-0.60, -0.56)
		67.5	-0.58 (-0.60, -0.56)
		72.5	-0.60 (-0.63, -0.58)
		77.5	-0.62 (-0.65, -0.59)
		82.5	-0.61 (-0.65, -0.56)
		87.5	-0.54 (-0.61, -0.46)
		92.5	-0.40 (-0.57, -0.23)
		97.5	-0.22 (-0.69, 0.25)
	Male	47.5	-0.41 (-0.47, -0.35)
		52.5	-0.55 (-0.60, -0.50)
		57.5	-0.62 (-0.67, -0.58)
		62.5	-0.65 (-0.69, -0.60)
		67.5	-0.65 (-0.70, -0.60)
		72.5	-0.64 (-0.70, -0.58)
		77.5	-0.59 (-0.67, -0.51)
		82.5	-0.49 (-0.61, -0.36)
		87.5	-0.37 (-0.59, -0.15)
		92.5	-0.23 (-0.76, 0.30)
		97.5	-0.11 (-1.60, 1.40)
	Female	47.5	-0.69 (-0.73, -0.66)
		52.5	-0.68 (-0.70, -0.65)
		57.5	-0.65 (-0.68, -0.63)
		62.5	-0.60 (-0.62, -0.58)
		67.5	-0.55 (-0.57, -0.53)
		72.5	-0.53 (-0.56, -0.50)
		77.5	-0.52 (-0.56, -0.49)
		82.5	-0.49 (-0.54, -0.44)
		87.5	-0.41 (-0.49, -0.33)

Measure	Sex	Age Group	Local Drift (% per year)
		92.5	-0.27 (-0.45, -0.10)
		97.5	-0.11 (-0.59, 0.38)
	Both	47.5	-0.55 (-0.57, -0.52)
		52.5	-0.58 (-0.60, -0.56)
		57.5	-0.59 (-0.61, -0.57)
		62.5	-0.58 (-0.60, -0.56)
		67.5	-0.57 (-0.59, -0.55)
		72.5	-0.60 (-0.62, -0.57)
		77.5	-0.62 (-0.65, -0.59)
		82.5	-0.60 (-0.65, -0.56)
		87.5	-0.53 (-0.61, -0.46)
		92.5	-0.40 (-0.57, -0.22)
		97.5	-0.22 (-0.71, 0.27)
YLDs	Male	47.5	-0.41 (-0.47, -0.36)
		52.5	-0.55 (-0.60, -0.51)
		57.5	-0.63 (-0.67, -0.58)
		62.5	-0.65 (-0.69, -0.60)
		67.5	-0.65 (-0.70, -0.60)
		72.5	-0.64 (-0.70, -0.58)
		77.5	-0.59 (-0.68, -0.51)
		82.5	-0.49 (-0.62, -0.36)
		87.5	-0.37 (-0.61, -0.14)
		92.5	-0.24 (-0.80, 0.33)
		97.5	-0.11 (-1.73, 1.53)
	Female	47.5	-0.68 (-0.71, -0.65)
		52.5	-0.66 (-0.69, -0.64)
		57.5	-0.64 (-0.66, -0.62)
		62.5	-0.59 (-0.61, -0.57)
		67.5	-0.54 (-0.57, -0.52)
		72.5	-0.53 (-0.55, -0.50)
		77.5	-0.52 (-0.55, -0.48)
		82.5	-0.49 (-0.54, -0.44)
		87.5	-0.41 (-0.50, -0.32)

Measure	Sex	Age Group	Local Drift (% per year)
		92.5	-0.28 (-0.47, -0.08)
		97.5	-0.12 (-0.67, 0.44)

Abbreviations: CI, Confidence Intervals. Local drift represents the annual percentage change in age-specific rates for each age group.

Supplementary Table S4. Period rate ratios from age-period-cohort analysis of low back pain incidence, prevalence, and YLDs among Chinese adults aged 45 years and older

Measure	Sex	Period	Period RR (95% CI)
Incidence	Both	1,992.5	1.105 (1.094, 1.115)
		1,997.5	1.029 (1.021, 1.037)
		2,002.5	1.021 (1.014, 1.027)
		2,007.5	1.000 (1.000, 1.000)
		2,012.5	0.983 (0.977, 0.989)
		2,017.5	0.972 (0.964, 0.979)
		2,022.5	0.928 (0.919, 0.937)
	Male	1,992.5	1.076 (1.052, 1.101)
		1,997.5	1.027 (1.009, 1.046)
		2,002.5	1.024 (1.010, 1.038)
		2,007.5	1.000 (1.000, 1.000)
		2,012.5	0.978 (0.965, 0.991)
		2,017.5	0.960 (0.944, 0.977)
		2,022.5	0.905 (0.884, 0.926)
	Female	1,992.5	1.115 (1.107, 1.123)
		1,997.5	1.024 (1.018, 1.031)
		2,002.5	1.016 (1.011, 1.021)
		2,007.5	1.000 (1.000, 1.000)
		2,012.5	0.989 (0.984, 0.994)
		2,017.5	0.983 (0.977, 0.988)
		2,022.5	0.948 (0.941, 0.955)
Prevalence	Both	1,992.5	1.122 (1.113, 1.131)
		1,997.5	1.033 (1.026, 1.040)
		2,002.5	1.024 (1.018, 1.029)
		2,007.5	1.000 (1.000, 1.000)
		2,012.5	0.980 (0.975, 0.985)
		2,017.5	0.966 (0.960, 0.972)
		2,022.5	0.921 (0.913, 0.928)
	Male	1,992.5	1.073 (1.050, 1.098)
		1,997.5	1.028 (1.010, 1.046)
		2,002.5	1.025 (1.011, 1.039)

Measure	Sex	Period	Period RR (95% CI)
		2,007.5	1.000 (1.000, 1.000)
		2,012.5	0.977 (0.964, 0.990)
		2,017.5	0.957 (0.941, 0.974)
		2,022.5	0.901 (0.881, 0.922)
		1,992.5	1.141 (1.131, 1.151)
		1,997.5	1.028 (1.020, 1.036)
		2,002.5	1.019 (1.013, 1.026)
	Female	2,007.5	1.000 (1.000, 1.000)
		2,012.5	0.985 (0.979, 0.991)
		2,017.5	0.978 (0.971, 0.985)
		2,022.5	0.940 (0.933, 0.948)
		1,992.5	1.118 (1.109, 1.127)
		1,997.5	1.032 (1.025, 1.039)
		2,002.5	1.023 (1.018, 1.029)
	Both	2,007.5	1.000 (1.000, 1.000)
		2,012.5	0.978 (0.973, 0.983)
		2,017.5	0.965 (0.959, 0.971)
		2,022.5	0.921 (0.914, 0.928)
		1,992.5	1.071 (1.046, 1.097)
		1,997.5	1.028 (1.010, 1.047)
		2,002.5	1.024 (1.010, 1.038)
YLDs	Male	2,007.5	1.000 (1.000, 1.000)
		2,012.5	0.974 (0.961, 0.987)
		2,017.5	0.955 (0.938, 0.972)
		2,022.5	0.901 (0.880, 0.923)
		1,992.5	1.137 (1.126, 1.148)
		1,997.5	1.027 (1.019, 1.035)
		2,002.5	1.019 (1.012, 1.026)
	Female	2,007.5	1.000 (1.000, 1.000)
		2,012.5	0.984 (0.977, 0.990)
		2,017.5	0.977 (0.969, 0.984)
		2,022.5	0.941 (0.932, 0.949)

Measure	Sex	Period	Period RR (95% CI)

Abbreviations: RR, Rate Ratio; CI, Confidence Intervals. Period effects represent the relative risk of each calendar period compared to the reference period (2007.5).

Supplementary Table S5. Cohort rate ratios from age-period-cohort analysis of low back pain incidence, prevalence, and YLDs among Chinese adults aged 45 years and older

Measure	Sex	Birth Cohort	Cohort RR (95% CI)
Incidence	Both	1,895	1.113 (0.856, 1.447)
		1,920	1.096 (1.084, 1.108)
		1,935	1.000 (1.000, 1.000)
		1,950	0.923 (0.917, 0.929)
		1,965	0.850 (0.843, 0.857)
		1,975	0.816 (0.806, 0.826)
	Male	1,895	1.110 (0.549, 2.243)
		1,920	1.090 (1.064, 1.116)
		1,935	1.000 (1.000, 1.000)
		1,950	0.904 (0.891, 0.916)
		1,965	0.831 (0.817, 0.844)
		1,975	0.819 (0.799, 0.838)
	Female	1,895	1.069 (0.882, 1.296)
		1,920	1.076 (1.066, 1.085)
		1,935	1.000 (1.000, 1.000)
		1,950	0.926 (0.921, 0.931)
		1,965	0.846 (0.840, 0.852)
		1,975	0.800 (0.792, 0.809)
Prevalence	Both	1,895	1.139 (0.922, 1.407)
		1,920	1.105 (1.094, 1.115)
		1,935	1.000 (1.000, 1.000)
		1,950	0.916 (0.910, 0.921)
		1,965	0.838 (0.832, 0.844)
		1,975	0.802 (0.793, 0.811)
	Male	1,895	1.111 (0.562, 2.194)
		1,920	1.094 (1.068, 1.121)
		1,935	1.000 (1.000, 1.000)
		1,950	0.901 (0.889, 0.914)
		1,965	0.834 (0.821, 0.848)
		1,975	0.829 (0.809, 0.849)
		1,895	1.089 (0.876, 1.353)

Measure	Sex	Birth Cohort	Cohort RR (95% CI)
	Female	1,920	1.083 (1.072, 1.095)
		1,935	1.000 (1.000, 1.000)
		1,950	0.915 (0.909, 0.921)
		1,965	0.824 (0.818, 0.831)
		1,975	0.769 (0.759, 0.780)
YLDs	Both	1,895	1.140 (0.912, 1.424)
		1,920	1.104 (1.094, 1.115)
		1,935	1.000 (1.000, 1.000)
		1,950	0.917 (0.912, 0.922)
		1,965	0.840 (0.834, 0.845)
		1,975	0.804 (0.796, 0.812)
	Male	1,895	1.112 (0.531, 2.332)
		1,920	1.095 (1.069, 1.122)
		1,935	1.000 (1.000, 1.000)
		1,950	0.902 (0.889, 0.914)
		1,965	0.834 (0.821, 0.847)
		1,975	0.828 (0.809, 0.847)
	Female	1,895	1.093 (0.851, 1.402)
		1,920	1.083 (1.071, 1.095)
		1,935	1.000 (1.000, 1.000)
		1,950	0.917 (0.910, 0.923)
		1,965	0.827 (0.820, 0.834)
		1,975	0.773 (0.763, 0.784)

Abbreviations: RR, Rate Ratio; CI, Confidence Intervals. Cohort effects represent the relative risk of each birth cohort compared to the reference cohort (1935).

Supplementary Table S6. Attributable YLD numbers, truncated age-standardized attributable YLD rates, and population attributable fractions for major modifiable risk factors of low back pain among Chinese adults aged 45 years and older, 1990–2023

	YLDs (×10⁴)			Share (%)		PAF (%)		EAPC (95% CI)		
	1990	2023	% change	1990	2023	1990	2023	Both	Female	Male
Occupational ergonomic factors	70.97	125.70	77.1	42.12	38.37	17.96	16.63	-0.81 (-0.87, -0.75)	-0.63 (-0.71, -0.55)	-1.10 (-1.16, -1.04)
High BMI	17.39	66.93	284.9	10.32	20.43	4.30	8.46	1.43 (1.33, 1.53)	1.34 (1.21, 1.47)	1.53 (1.47, 1.58)
Smoking	80.16	134.93	68.3	47.56	41.19	19.92	17.06	-0.97 (-1.05, -0.88)	-2.07 (-2.16, -1.97)	-0.64 (-0.72, -0.55)

Abbreviations: YLDs, Years Lived with Disability; PAF, Population Attributable Fraction; EAPC, Estimated Annual Percentage Change; CI, Confidence Interval. Notes: YLDs are presented in 10,000 person-years for both sexes combined. % change refers to the relative change in the absolute number of attributable YLDs. Share denotes the proportion of total LBP YLDs attributable to each risk factor among adults aged ≥45 years. PAF was calculated for all adults aged 45+ years combined. EAPCs were derived from log-linear regression of truncated age-standardized rates.

Supplementary Table S7. Observed and projected age-standardized incidence, prevalence, and YLD rates of low back pain among Chinese adults aged 45 years and older, by sex, 1990–2040

Outcome	Year	Data type	Both sexes	Male	Female
Incidence	1,990	Observed	6308.52 (6305.16, 6311.87)	4681.97 (4677.65, 4686.29)	7889.15 (7884.02, 7894.29)
	1,995		5610.10 (5607.17, 5613.03)	4322.14 (4318.37, 4325.91)	6871.35 (6866.88, 6875.83)
	2,000		5569.18 (5566.48, 5571.87)	4299.91 (4296.46, 4303.36)	6822.39 (6818.25, 6826.52)
	2,005		5434.39 (5431.93, 5436.85)	4178.03 (4174.91, 4181.16)	6679.84 (6676.03, 6683.64)
	2,010		5330.81 (5328.60, 5333.02)	4067.48 (4064.70, 4070.26)	6578.07 (6574.63, 6581.50)
	2,015		5238.14 (5236.16, 5240.13)	3983.68 (3981.19, 3986.16)	6464.85 (6461.76, 6467.94)
	2,020		5173.89 (5172.06, 5175.72)	3866.98 (3864.71, 3869.25)	6440.05 (6437.20, 6442.91)
	2,023		4751.08 (4749.37, 4752.79)	3582.21 (3580.07, 3584.35)	5873.48 (5870.83, 5876.13)
	2,025	Projected	4568.97 (4435.05, 4702.89)	3459.06 (3354.51, 3563.61)	5617.20 (5439.15, 5795.25)
	2,030		4061.36 (3538.78, 4583.95)	3109.94 (2730.36, 3489.52)	4936.27 (4243.31, 5629.23)
	2,035		3605.65 (2633.55, 4577.75)	2805.13 (2096.78, 3513.49)	4320.09 (3048.13, 5592.05)
	2,040		3197.07 (1781.75, 4612.39)	2537.12 (1489.46, 3584.78)	3766.30 (1945.46, 5587.14)
Prevalence	1,990	Observed	15324.23 (15318.93, 15329.52)	11035.87 (11029.12, 11042.63)	19423.25 (19415.13, 19431.37)
	1,995		13342.52 (13337.95, 13347.09)	10191.41 (10185.52, 10197.31)	16366.42 (16359.45, 16373.38)
	2,000		13259.58 (13255.39, 13263.78)	10148.14 (10142.77, 10153.50)	16281.87 (16275.42, 16288.31)
	2,005		12912.43 (12908.61, 12916.25)	9864.13 (9859.29, 9868.96)	15893.61 (15887.71, 15899.51)
	2,010		12630.83 (12627.40, 12634.25)	9608.19 (9603.88, 9612.49)	15581.02 (15575.71, 15586.34)
	2,015		12395.11 (12392.05, 12398.18)	9404.66 (9400.83, 9408.49)	15291.66 (15286.89, 15296.42)
	2,020		12267.10 (12264.28, 12269.92)	9167.04 (9163.53, 9170.54)	15240.66 (15236.27, 15245.05)
	2,023		11215.80 (11213.18, 11218.42)	8488.61 (8485.32, 8491.91)	13805.41 (13801.36, 13809.47)
	2,025	Projected	10783.80 (10443.13, 11124.46)	8215.37 (7958.81, 8471.93)	13165.83 (12682.07, 13649.59)
	2,030		9619.86 (8248.50, 10991.21)	7417.90 (6486.40, 8349.41)	11556.27 (9641.59, 13470.96)
	2,035		8568.68 (5999.34, 11138.01)	6720.60 (4975.61, 8465.58)	10088.99 (6576.62, 13601.36)
	2,040		7620.89 (3865.09, 11376.69)	6105.88 (3514.32, 8697.44)	8763.44 (3752.83, 13774.04)
YLD	1,990	Observed	1684.94 (1683.23, 1686.65)	1223.06 (1220.93, 1225.19)	2131.02 (2128.36, 2133.67)
	1,995		1473.46 (1471.98, 1474.93)	1134.10 (1132.24, 1135.96)	1802.46 (1800.19, 1804.73)
	2,000		1463.64 (1462.28, 1465.01)	1127.45 (1125.74, 1129.15)	1793.00 (1790.90, 1795.11)
	2,005		1427.57 (1426.32, 1428.82)	1097.68 (1096.13, 1099.24)	1752.56 (1750.63, 1754.49)
	2,010		1393.20 (1392.08, 1394.32)	1065.45 (1064.06, 1066.85)	1715.03 (1713.28, 1716.77)
	2,015		1365.99 (1364.98, 1366.99)	1041.99 (1040.73, 1043.24)	1681.53 (1679.96, 1683.10)
	2,020		1357.14 (1356.20, 1358.07)	1019.75 (1018.59, 1020.90)	1682.12 (1680.67, 1683.57)

Outcome	Year	Data type	Both sexes	Male	Female
	2,023		1238.05 (1237.18, 1238.93)	943.19 (942.10, 944.28)	1519.40 (1518.05, 1520.74)
	2,025		1190.26 (1151.12, 1229.39)	911.68 (882.67, 940.69)	1448.89 (1394.06, 1503.72)
	2,030	Projected	1062.88 (900.76, 1224.99)	822.71 (717.09, 928.33)	1272.97 (1048.80, 1497.14)
	2,035		947.77 (642.59, 1252.94)	745.08 (547.17, 942.98)	1112.38 (698.85, 1525.90)
	2,040		843.90 (396.71, 1291.10)	676.73 (382.77, 970.69)	967.14 (375.70, 1558.58)

Abbreviations: UI, Uncertainty Intervals (for observed values, from GBD draws); CrI, Credible Intervals (for projected values, from BAPC posterior distributions). Values are age-standardized rates per 100,000 population, standardized to the GBD world standard population restricted to ages 45+. Observed values (1990–2023) are from GBD 2023; projected values (2024–2040) are from Bayesian age-period-cohort (BAPC) models implemented via the BAPC R package with INLA. Years 1990–2020 and 2024–2040 are shown at 5-year intervals; 2023 is included as the last observed year.
